# Biomolecular Profiling by MALDI-TOF Mass Spectrometry in Food and Beverage Analyses

**DOI:** 10.3390/ijms232113631

**Published:** 2022-11-07

**Authors:** Marek Šebela

**Affiliations:** Department of Biochemistry, Faculty of Science, Palacký University, Šlechtitelů 27, CZ-783 71 Olomouc, Czech Republic; marek.sebela@upol.cz

**Keywords:** adulteration, classification, differentiation, food, mass spectrometry, MALDI, marker, milk, oil, protein

## Abstract

Matrix-assisted laser desorption/ionization time-of-flight mass spectrometry (MALDI-TOF MS) has frequently been applied to the analysis of biomolecules. Its strength resides not only in compound identification but particularly in acquiring molecular profiles providing a high discriminating power. The main advantages include its speed, simplicity, versatility, minimum sample preparation needs, and a relatively high tolerance to salts. Other benefits are represented by the possibility of automation, high throughput, sensitivity, accuracy, and good reproducibility, allowing quantitative studies. This review deals with the prominent use of MALDI-TOF MS profiling in food and beverage analysis ranging from the simple detection of sample constituents to quantifications of marker compounds, quality control, and assessment of product authenticity. This review summarizes relevant discoveries that have been obtained with milk and milk products, edible oils, wine, beer, flour, meat, honey, and other alimentary products. Marker molecules are specified: proteins and peptides for milk, cheeses, flour, meat, wine and beer; triacylglycerols and phospholipids for oils; and low-molecular-weight metabolites for wine, beer and chocolate. Special attention is paid to sample preparation techniques and the combination of spectral profiling and statistical evaluation methods, which is powerful for the differentiation of samples and the sensitive detection of frauds and adulterations.

## 1. Introduction

Matrix-assisted laser/desorption ionization (MALDI) is one of the soft ionization techniques in mass spectrometry (MS). It has found its long-lasting application in analyzing various biomolecules since its introduction in the 1980s [1]. Its ability to ionize proteins without undesired fragmentation was recognized very early [2,3]. Mass spectrometers equipped with MALDI typically contain a time-of-flight (TOF) mass analyzer, which can be upgraded to a higher resolution by the presence of a reflector (reflectron) part. The principle of TOF function fits well to the pulsed character of MALDI [4]. In order to measure mass spectra, the sample is mixed with a matrix solution and applied onto the target plate for crystallization [1]. The matrix is a chemical compound, which readily absorbs in crystals the laser energy at a given wavelength to facilitate the sample ionization process (an example is 2,5-dihydroxybenzoic acid, i.e., DHB).

In addition to proteins and peptides, MALDI-TOF MS has been shown suitable to measure glycans, lipids, oligonucleotides, metabolites, or synthetic polymers [1]. In proteomics, MALDI is the basis of several protein identification approaches, including peptide mass fingerprinting [5,6] or peptide sequencing on TOF/TOF instruments, which allow for the controlled fragmentation of peptides yielding readable sequence information [7,8]. Triacylglycerols (TAGs) can be identified by their mass in fingerprint spectra, but a detailed structural analysis with the identification of positional isomers requires a high-energy collision-induced dissociation with fragmentations of the fatty acid substituents performed on TOF/TOF instruments [9]. Common omics approaches with extractions and liquid chromatographic separations of complex biomolecular mixtures prior to MS measurements are not easily transferrable to a daily routine due to laborious sample preparation procedures and time-consuming data acquisition. On the other hand, the use of MALDI-TOF MS molecular profiling is simple, straightforward, rapid (there is no need for a special sample pre-treatment or separation), reproducible, sensitive, and robust [10]. Its versatility resides in its applicability from low molecular weight compounds to polypeptides for both hydrophilic and hydrophobic molecules. There are also other advantages, namely, a high tolerance to impurities, high throughput, easy automation, and cost effectiveness.

Consumers expect that they always purchase food of a high and stable quality, which is safe to their health and not allergenic or harmful. Therefore, there is a need for sensitive, fast, reliable, and cost-effective methods to prevent frauds and adulterations that could successfully complement or replace those that are already in use [11]. In this context, the traceability of specific markers is essential to monitor identity and unique quality traits. In milk analysis, the resolution of MALDI-TOF MS is high enough to separate protein peaks differing by a few mass units in the mass range of 2–25 kDa for detecting the presence of adulteration [12]. Similarly, the high resolution and accuracy provided by measurements in the reflector ion mode allow specific TAGs to be recognized by the direct analysis of oil samples. The rate of adulteration can be quantified based on signal intensities of marker biomolecules [13]. However, the application of MALDI for quantitative purposes is hampered by several intrinsic pitfalls. Typically, a non-homogeneous distribution of the analyte in the sample-matrix cocrystals influences the shot-to-shot and sample-to-sample reproducibility. Moreover, signal intensities of similar compounds are influenced by the structure; peak height decreases with increasing molecular mass of TAG standards at the same concentrations [14]. However, careful optimizations of the experimental conditions (spectra are averaged from replicates and accumulated from different sample spot positions) or the use of internal standards can lead to experimental quantitative values being in good agreement with the real composition.

When two or more chemically similar mixtures are combined, a single biomarker may not be sufficient to detect an adulteration [15]. Mass profiles are thus used, which show highly reproducible and species-specific patterns with strong discriminating power. The molecular profiling of biological samples by MALDI-TOF MS has been widely used in combination with software tools for data analysis, such as a principal component analysis (PCA), hierarchical clustering, and various supervised learning techniques for constructing predictive models [16]. The processing by multivariate analysis applied to MALDI spectral profiles simplifies the data complexity but retains significant sample patterns (i.e., reduces the dimensionality of datasets to the features that contain the most information). PCA is the most common approach for first attempts to classify samples into distinct groups. However, it may provide ambiguous classifications. Different sample sizes in the same study may yield alternative results, and PCA is also sensitive to the scaling of the variables. On the other hand, supervised models, for example partial least squares discriminant analysis (PLS-DA), permit multicategory classification with features extracted from complex data [17]. However, the classification results are highly dependent on optimizing the processing parameters.

This review article summarizes MALDI-TOF MS profiling experiments that have been performed for a food and beverage analysis. The following alimentary products were characterized in this way and are thus mentioned in the text: milk and milk products, edible oils, wine, beer, flour, meat and meat products, potatoes, honey, coffee beans, and chocolate. The milk products included cheeses, curd, kefir, and yoghurt. Vegetable oils included particularly virgin olive oil, but also various seed oils and animal fats. Proteins and peptides were used as marker compounds of different milks (e.g., bovine, ovine, or caprine milk), whereas TAGs and phospholipids were profiled and identified for a discriminating purpose in oils. Generally, the fingerprint mass spectra were acquired after optimizing the sample preparation procedure with the selection of the most proper matrix compound, solvents, and deposition techniques for crystallization on the target. The obtained data were processed according to the needs ranging from a simple detection of sample constituents to quantifications of marker compounds, quality control, and assessment of product authenticity requiring sophisticated statistical data processing. Similar topics were reviewed from a different perspective (food adulteration) in 2021 [18].

## 2. Milk and Milk Products

**Figure 1:**
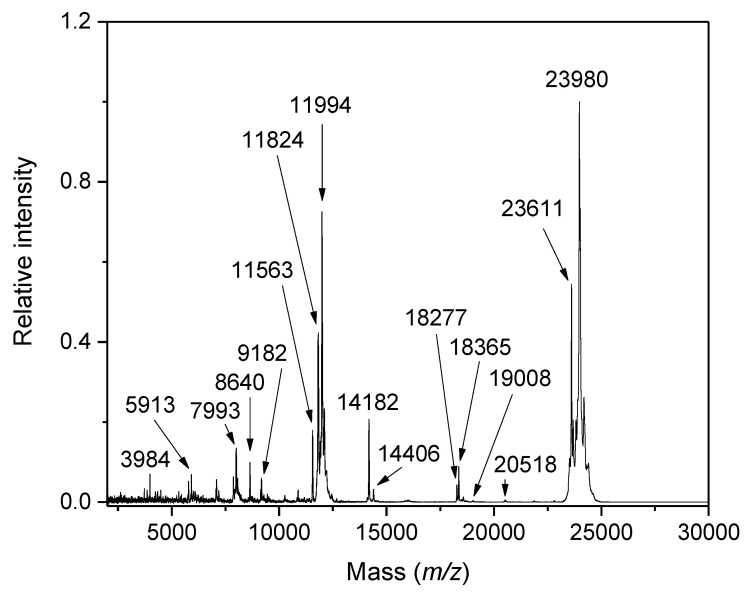
MALDI-TOF MS of milk. This sample of aseptically packed fresh milk was measured using an ultrafleXtreme instrument (Bruker Daltonics). The depicted spectrum was acquired in the linear positive ion mode with a stainless-steel target and sinapinic acid (SA) as a matrix according to Fanton et al. [27]. Many peptides were detected at masses below 6 kDa such as that at *m/z* 3984, which is a fragment of α_S1_-casein [28]. Major protein peaks refer to γ_3_-casein (*m/z* 11,563), γ_2_-casein (*m/z* 11,824), α-lactalbumin (*m/z* 14,182), β-lactoglobulin (*m/z* 18,277 and 18,365—two variants), κ-casein (*m/z* 19,008), γ_1_-casein (*m/z* 20,518), α_S1_-casein (*m/z* 23,611), and β-casein (*m/z* 23,980). The content is author´s own work.

Milk is a heterogeneous mixture and highly nutritional foodstuff. It is an emulsion of fats in a liquid called milk plasma. The liquid contains water, suspended proteins (caseins), soluble proteins (whey proteins), sugars (mainly lactose), and minerals. Many other compounds such as vitamins appear in low quantities [19]. The whey fraction of donkey milk contains α-lactalbumin and β-lactoglobulin as major proteins but also lactoferrin, lysozyme, and serum albumin [20]. The presence of microorganisms negatively influences the shelf life of raw milk. Thus, several kinds of thermal treatments such as pasteurization, in-container sterilization and ultra-high temperature (UHT) processing are employed in the dairy industry to preserve milk quality for a prolonged time. Pasteurization is performed by mild heating at a minimum of 72 °C for 15 s, which destroys most pathogenic and spoilage microbes. Conversely, UHT treatment (in a direct or indirect way) is conducted at about 140 °C for a few seconds, and its effects impact all microorganisms, including bacterial spores [21]. These stabilizing thermal treatments of milk induce chemical changes, which are dependent on the applied heat load. Milk and milk products need to be controlled during production to preserve their quality, safety, stability, and nutritional value. This is also motivated by customers’ requirements for consistent product behavior [22]. Milk components undergo changes and modifications induced by processing steps and storage. Hence, mass spectrometry has become a powerful tool for quality control. Soft ionization techniques coupled with liquid chromatography (LC)-based separations represent a standard analytical approach for lipids in milk [22]. Lipid oxidation resulting in the formation of hydroperoxides and secondary oxidation products (e.g., carbonyl compounds such as malondialdehyde) is a major cause of deterioration of milk quality. Protein glycation is a result of non-enzymatic reaction of amino groups with carbonyl groups of reducing sugars, for example lactose or lactulose. It originates from the Maillard reaction [23]. MALDI-TOF MS is very effective in identifying milk proteins (Figure 1) and allows for the assessment of the extent of protein modifications, which appear as a consequence of raw milk processing [24]. It was utilized to analyze milk samples from different cow breeds, different lactation stages of the same breed, and bulk milk from various geographical areas and thermal treatments. Differences in abundances of specific protein ions registered in milk samples processed by thermal treatments were applied in a multivariate statistical analysis for clustering [25]. Both pasteurization and sterilization of raw milk were compared regarding the impact of protein denaturation by heat on the MALDI-TOF spectral profile, indicating that especially direct-steam UHT heating leads to the disappearance of low mass protein signals [26].

The most common illegal practices related to milk include the selling of skimmed milk instead of whole milk and the dilution of milk with water (not addressed in this review), the addition of dried milk products into fresh milk, and milk adulteration with a lower value milk, e.g., ewe milk with cow milk. Detection techniques such as liquid chromatography, immunoassays, polymerase chain reaction (PCR), and electrophoresis have been reviewed [29]. MALDI-TOF MS profiling analyses of milk and milk products can be divided into two groups based on whether proteins or peptides are measured.

Different proteins (such as caseins, α-lactalbumin, and β-lactoglobulins) can be identified from MALDI mass spectra of milk by their specific molecular masses. The mass differences based on individual amino acid sequences are useful for differentiating the origin of milk. A detection limit below 5% was found for a fraudulent addition of cow milk into water buffalo milk. Similarly, the thermal-induced glycation by lactose of α-lactalbumin and β-lactoglobulins has been introduced as a marker to detect the presence of powdered milk in samples of fresh milk [12]. The same detection limit of 5% for the presence of bovine milk in ovine and caprine milk was achieved using a different approach based on measuring MALDI-TOF mass spectra of milk protein tryptic digests when α-cyano-4-chlorocinnamic acid (CClCA) was applied as a matrix [30]. This strategy was found to be beneficial for distinguishing milk from closely related animal species such as sheep and goat. Cow-specific and goat-specific peptide markers, attributed to their proteins of origin by database searches, were used to monitor the authenticity of milk samples. Not all of them were visible in the mass spectra acquired at a low level of adulteration. However, the others were confirmed as valid and reliable markers showing a near linear dependence of their signal intensities on the percentage of the adulterant milk [30]. Hierarchical clustering dendrograms and PCA plots were found very useful to detect adulterations of donkey and goat milk (e.g., by cow milk) reliably down to 0.5% of the adulterant [31]. For this purpose, the whole peptide and protein MALDI-TOF spectral profiles were acquired in the mass range of *m/z* 2000–25,000 with sinapinic acid (SA) matrix and processed for statistical analyses.

Milk proteins were measured by MALDI-TOF MS to demonstrate differences between bovine and water buffalo milk and a mozzarella cheese made from either [32]. MALDI-TOF MS has been introduced for this purpose as an efficient alternative to electrophoretic and chromatographic methods based on differentiating peptide/protein patterns. The measured molecular masses of intact proteins indicated the presence of a protein species (*m/z* 15,791), which clearly discriminated buffalo and cow milk and was found applicable to authenticate buffalo mozzarella by its presence. Later it was identified as a β-casein fragment [33]. This protein signal was useful to quantify the content of bovine milk added as an adulterant [32]. Whey proteins, namely, α-lactalbumin and β-lactoglobulins, were used as protein markers to identify adulterations of fresh water buffalo mozzarella cheese with cheaper cow and ewe milk. For example, the mass difference between the buffalo and ovine counterpart proteins is around 100 Da. A linear response was found for the molecular ion intensity and abundance of the whey proteins, which allowed the content of the added adulterant to be determined [34]. Peak pattern differences between Italian cheeses made exclusively from cow or ewe milk were demonstrated using MALDI-TOF MS with SA as a matrix. The measured peak area ratio for ovine and bovine γ_2_-casein as specific proteins markers could be used for a straightforward quantitative analysis of possible adulteration of the ewe cheese by cow milk [27]. Possible adulterations of donkey milk by milk from ruminants was performed by monitoring the presence of specific peaks of the most abundant whey proteins, α-lactalbumin and β-lactoglobulins, in MALDI-TOF mass spectra acquired using SA as a matrix [35]. Specific protein masses were discernable down to a concentration of the adulterant of 0.5–2%. The relative quantification of adulteration in model binary milk mixtures was done via the areas of α-lactalbumin peaks in the spectra (assuming that they were proportional to its relative concentration), providing a good correlation with the real adulteration level, which was comparable to that achievable by more laborious analytical methods.

Another strategy for the protein profiling of milk was based on MALDI-TOF MS of proteotypic peptides in tryptic digests of the casein fraction isolated by isoelectric precipitation. These “signature peptides” have successfully been introduced to differentiate milk samples from four different species: cow, sheep, goat, and water buffalo [36]. Most of them originated from α_S1_- and α_S2_-caseins but were also from β-casein. Synthetic peptides related to the species-specific α_S1_-casein sequences proved to be useful internal standards for quantification. The presence of bovine and ovine milk as an adulterant in water buffalo milk was detected with a limit of 0.5%. For caprine milk it was 1%. The absolute amount of α_S1_-casein (0.02–0.2 ng/µL) could be converted into adulteration percentage, but this number was not exactly equivalent to the percentage of the adulterant milk, as the content of α_S1_-casein was found to be species- and sample-specific [36]. The peptide fraction of milk was analyzed by MALDI-TOF MS after chromatography with magnetic beads carrying immobilized copper (II) ions for unspecific enrichment [28]. The most prominent signals in the surveyed mass area belonged to α_S1_-casein fragments (*m/z* 3984 and 4340). This procedure allowed for studies on changes induced by heat treatment as well as storage. Compared to raw milk, several peptide signals newly appeared upon heating at 120 °C resulting from casein fragmentation. The prolonged storage of UHT milk provided an increase in the *m/z* 4340 signal, which was elucidated by the presence of milk protease activity. Cow-specific tryptic peptides from milk proteins were clearly distinguishable by measuring extracts from Grana Padano and Pecorino Romano cheeses made from cow and sheep milk, respectively [30]. Water buffalo milk and curd samples were also analyzed for the presence of specific casein variants to differentiate genuine Italian milk from that imported from abroad [37]. The reason for this variants resides in cross breeding, which did not occur in Italy due to the isolation of the water buffalo population. The isoelectric casein was digested by trypsin in native as well as dephoshorylated (by alkaline phosphatase) forms, and several α_S1_-casein- and β-casein-derived peptides were found to be good milk origin markers. The study additionally involved the selective chromatographic enrichment of casein phosphopeptides on hydroxyapatite. Two phosphopeptides were demonstrated as suitable markers to find foreign milk as an adulterant in products sold under the protected designation of origin label. Similarly, a β-casein phosphopeptide f1-28 allowed a buffalo mozzarella cheese to be found adulterated by bovine milk after casein digestion by plasmin [37]. Adulterations of water buffalo, goat, and ovine milk by added bovine milk (1–50%, *v/v*) were also detected and quantified using a combined MALDI-TOF MS analysis of proteins and peptides [38]. The authors additionally determined protein and peptide markers of a thermal treatment of milk (pasteurized, UHT, and powdered milk), which potentially allow one to detect fraudulent raw milk samples. Skimmed milk samples were measured directly after a dilution to obtain protein profiles with an SA matrix, or diluted and ultrafiltered (optionally also enriched in peptides using ZipTipC18 reversed-phase pipetted tips) prior to acquiring the corresponding peptide profile spectra with α-cyano-4-hydroxycinnamic acid (CHCA) as a matrix. The experimental data were subjected to PCA. PLS regression plots for training samples showed linear correlations between known adulteration levels and those predicted from the PCA-processed spectral data, which allowed an accurate assessment of test samples.

Camembert is a soft cheese of high humidity. Its ripening is accompanied by the proteolysis of casein mediated by milk enzymes such as plasmin and enzymes from the microbial cultures related to the technology. This proteolysis releases bioactive peptides with a positive impact on human health and body functions. MALDI-TOF MS allowed the production of bioactive peptides to be monitored in camembert made from raw or pasteurized milk, with or without adding a culture of *Lactobacillus rhamnosus*, and ripened for a period of up to 50 days [39]. The observed peptides were assigned based on their masses, and the collected results over the ripening time period for all cheese variants were statistically processed (using PLS-DA). As a result, the cheeses made with raw milk showed better differentiation. Namely, the bioactive peptides derived from β-casein had the highest discriminating power. In another study, MALDI-TOF MS fingerprinting of protein signals (whey proteins, caseins, and their degradation fragments) followed by data processing with statistical methods (PCA, PLS) has been recommended for a routine check of ovine cheese (Greek feta) adulteration down to a 1% cow milk concentration [40]. Discrimination analyses of authentic feta cheeses, pure white cheeses, and a series of feta cheeses with different levels of adulteration were performed to build a supervised discrimination model. The developed model was applied to analyze yoghurt samples made with a pure ovine milk or adulterated with bovine milk [40]. MALDI-TOF MS was employed for evaluating the relationship between the content of the β-casein fragment (f193-209) and the sensory bitterness of an aged Cheddar cheese [41]. The MS quantification utilized a synthetic peptide internal standard. The analyzed samples were prepared from cheeses aged up to 270 days, and their β-casein peptide fragment contents correlated with the bitterness analyzed by a panel of trained tasters. Interestingly, the correlation of bitter peptide and sensory bitterness was found to be weaker with the progress of aging, which was elucidated by the presence of more bitter peptides at longer aging or a compound that masks the intensity of bitterness.

Kefir is a drink made from fermented milk, which is consumed for its health-promoting effects. Kefir phosphopeptides (including 14 multiphosphorylated ones with 2–5 phosphoserine residues) were enriched by their adsorption onto hydroxyapatite, identified by MALDI-TOF MS and sequenced by electrospray ionization tandem mass spectrometry (ESI–MS/MS) [42]. They are released from milk proteins (particularly β-casein) during fermentation, and some of them can efficiently bind bivalent cations such as Ca(II). Thus, upon consumption, they contribute to the bioavailability and resorption of minerals in the gastrointestinal tract. The same group has shown by MALDI-TOF MS profiling and quantification with an internal standard that the peptides released by the proteolysis of β-casein are increased in their level during the storage of UHT sterilized milk [43]. This is because of the activity of endogenous milk proteases and residual microbial proteases. A statistical evaluation based on the receiver operator characteristics (ROC) curve and area under the curve (AUC) values allowed the most suitable peptide markers of proteolysis to be identified. The best candidate for the detection of expired UHT milk samples, β-casein f192-206, provided a 100% sensitivity (true positive rate) and specificity (true negative rate), which was evaluated in a blind test (100% accuracy rate) [43]. Similarly, seven peptide markers differentiating between pasteurized and extended shelf life (ESL) milk were identified by MALDI-TOF MS profiling and relative quantification followed by statistical evaluations to define their cutoff levels for a prediction model [44]. The model showed an accuracy of 95% with test samples. The amino acid sequences of the majority of the marker peptides were determined by LC–ESI–MS/MS. They were derived from α_S1_-casein and β-casein by the action of milk cathepsins or plasmin. Another way of looking for marker peptides in milk was developed in connection with the use of surface-active maghemite particles, which bind peptides and proteins due to solvent-exposed Fe(III) sites [45]. A bound peptide fragment of α_S1_-casein identified by MALDI-TOF MS (*m/z* 4338) has been established as a marker of bovine mastitis disease. As demonstrated by molecular modelling, the binding mode of the peptide involves its carboxylic residues. Relative quantifications showed a correlation between the signal intensity and somatic cell counts in milk related to the inflammation, and they allowed a decision threshold value (normalized peak intensity) to be found for discrimination by logistic regression.

A method for high throughput screening of milk powder adulteration with vegetable oils and animal fat by MALDI-QTOF MS resided in determining spectral profiles of TAGs as low-molecular-weight compounds [46]. The analyzed samples were extracted by hexane and then measured. PCA analysis of the acquired data distinguished clearly the adulterated samples. Another fast and sensitive authentication method to distinguish bovine milk from non-dairy milks (e.g., coconut or soya milk) by MALDI-TOF MS lipid fingerprinting utilized super-DHB, a 9:1 mixture of DHB and 2-hydroxy-5-methoxybenzoic acid, as a matrix [47]. Milk samples were diluted with water and then mixed with the matrix on the target plate for crystallization. The most abundant peaks in the bovine and soy milk spectra were attributed to phosphatidylcholines, whereas coconut milk provided predominant signals of TAGs (it is higher in fat than whole dairy milk). PCA was used to check the discrimination power of the method. All types of analyzed milk were clearly separated in the corresponding groups. Concentration-dependent relationships between the relative abundances of marker peaks were found to be useful to evaluate quantitatively the composition of binary milk mixtures.

Binary and ternary mixtures of pasteurized bovine, caprine, and ovine milk were analyzed by MALDI-TOF MS of intact proteins followed by multivariate statistical analyses to explore the accuracy of this method for the detection and quantification of milk adulteration [48]. The measured protein profiles (whole spectra) were first base-line corrected, normalized, and then processed by PLS regression and nonlinear Kernel PLS regression to achieve calibration plots with the predicted versus real adulterant concentration. The obtained regression models showed low root mean square errors (RMSEs) of prediction of 2–4% for the ternary mixtures and 6–13% for the binary mixtures. Spectral peaks with the highest variable importance for the prediction scores included, for example, those of α-lactalbumin, β-lactoglobulin, and γ_2_- and γ_3_-casein [48]. A more recent paper on caprine and ovine milk adulteration with bovine milk also relied on MALDI-TOF MS and multivariate statistical analyses [49]. The analyzed samples originated from different geographical regions and were sampled in two lactation periods. Their number and complexity were further increased by performing serial experimental adulterations. Spectra acquisitions in the mass region of 500–4000 Da provided lower RMSE values of the prediction than those for 4–20 kDa. The RMSE numbers from regression calibration models could be decreased from more than 10% down to 3–6% by decreasing the variability of samples.

As can be seen from the above examples, MALDI-TOF fingerprint mass spectra for milk or milk product analysis are well obtainable with low-molecular-weight compounds (peptides, TAGs, phospholipids) as well as proteins. The measurement with peptides/phosphopeptides may provide higher sensitivity, and it is applicable for more analytical purposes. In addition to the information (qualitative or quantitative) on possible adulteration provided by protein, peptide, and lipid analysis, the presence of well-resolved and specific peptides indicates the mode of thermal treatment of milk, its storage conditions, veterinary issues, and geographical origin or evaluates the taste character (bitterness) of ripened cheeses. On the other hand, a minimum sample treatment is required when milk proteins are measured, whereas an extraction step is needed to analyze lipids. The proteolytic digestion followed by peptide purification and/or enrichment definitely represents a more time-consuming protocol.

## 3. Vegetable and Other Edible Oils; Fats

Vegetable and other edible oils are largely composed of TAGs, representing an important source of energy and nutrition. Their analysis is therefore focused on the TAG profile characterization and identification of constituent fatty acids. The analysis of these oils also addresses the problem of counterfeiting and adulteration, which is very important, especially in the case of extra virgin olive oil (EVOO) [13]. The most common analytical methods used for this purpose include high-performance liquid chromatography (HPLC), gas chromatography (GC), or methods coupled with MS such as HPLC–MS or GC–MS [50]. Fatty acids, usually originating from TAGs, are typically converted to their esters prior to analysis, which are less polar and thus more compatible with chromatographic systems. MS analysis of TAGs generally provides information on their molecular mass and constituent fatty acids. MALDI-TOF MS is a simple, accurate, and fast choice for the purpose of TAG profiling, although it cannot distinguish positional isomers [51]. The complete structural analysis of [M+Na]^+^ ions of TAGs by high-energy collision-induced dissociation with charge-remote fragmentations of the fatty acid substituents was performed using a MALDI-TOF/TOF instrument and 2,4,6-trihydroxyacetophenone (THAP) as a matrix [9]. The presence of specific fragments in the product ion spectra allowed positional isomer differentiation, except for determining the chirality at the *sn*-2 position. The authors pointed out a limitation given by the selection of precursor ions in a mass window of four units, which precluded analysis of TAGs differing by a double bond [9]. A MALDI-TOF/TOF instrumentation allowing the isolation of monoisotopic precursor ions with unit selectivity for high-energy collision-induced dissociation was used to overcome this limitation in investigations performed for a detailed structural analysis of TAGs and their isomers (except stereochemistry) in olive oil [52]. The DHB matrix with sodium trifluoroacetate as a cationizer as well as TAG and oil samples were dissolved in tetrahydrofuran (THF) [52]. It is worth mentioning here that TAGs are very detectable without using any matrix by laser desorption/ionization time-of-flight mass spectrometry (LDI-TOF). Sodium adduct peaks of TAGs were obtained in the spectra when an optimized protocol was used. Oil samples were dissolved in acetonitrile (ACN)/THF, 75:25, *v/v*, before deposition on a stainless-steel target [53].

DHB dissolved in acetone was found to be a suitable matrix for the analysis of pure TAGs, olive oil, and extracts from onion seeds [14]. Adding TAG samples to a layer of small DHB crystals provided a very good shot-to-shot reproducibility. Thus, the relative intensities of TAG sodium adduct peaks were useful for quantification, and the limit of detection was about 100 fmol for a trilaurin standard. A process of oxidative aging simulated for the TAGs in the onion extracts resulted in the formation of oxidized TAGs, as demonstrated by a mass difference of 16 Da. In another study, the TAG composition of olive, canola, castor, and vernonia oils was characterized by acquiring MALDI-TOF mass spectra using CHCA as a matrix dissolved in the mixture of ACN and THF for a fast evaporation during on-target crystallization. TAGs were detected as sodiated ions [54]. Special edible oils such as those from flax, grape, sesame seeds, hazelnuts, and walnuts were characterized for their TAG profiles in a similar way [55]. The registered TAG molecular complexity decreased as follows: flaxseed > walnut > sesame > grapeseed > hazelnut. The identified fatty acid moieties in the detected TAG molecules (e.g., the predominant linolenic acid in flaxseed oil and linoleic acid in the grapeseed and walnut oils) were consistent with previous literature data from other methods. Interestingly, the use of 1,2-distearoyl-3-palmitoylglycerol as an internal standard resulted in an observation that its sodiated ion signal is largely reduced in intensity in the presence of unsaturated TAGs, biasing accurate quantifications [55]. Positive ion MALDI-TOF mass spectra were acquired in the reflector to compare TAG profiles of two brands of cod liver oil [56]. The measurements were conducted with an optimized mass ratio of sample to matrix (CHCA was dissolved in a mixture of ACN and THF, 7:1, *v/v*). Sixty-four different TAGs were determined as [M + Na]^+^ signals in the *m/z* range of 700–1100, including those referring to the presence of polyunsaturated fatty acids such as eicosapentaenoic and docosahexaenoic acids [56]. The observation that the two predominant fatty acids from vernonia and castor oils, vernolic and ricinoleic acid, respectively, were converted partly into sodium carboxylates (soaps) in the presence of sodium acetate as a dopant compound and then provided sodiated ions in the spectra during MALDI-TOF MS measurements, led to the analysis of saponified vegetable oils [57]. The measured samples were prepared from palm kernel oil, palm oil, olive oil, soybean oil, vernonia oil, and castor oil. Two commercial soaps were additionally included. MALDI-TOF mass spectra were acquired with *meso*-tetrakis(pentafluorophenyl)porphyrin (F20TPP) as a matrix. This matrix is advantageous for low-molecular-weight analytes, as it does not produce interfering ions below *m/z* 500. Except for qualitative determination of the constituent fatty acids in the samples as sodiated sodium carboxylates, relative quantification data were also obtained using the determined relative response factors of individual fatty acids. The quantitative results were found consistent with previous GC–MS experiments [57]. It has been reported that the use of a target plate precoated with a thin layer of nitrocellulose reduced the in-source fragmentation of the measured TAGs [58]. DHB dissolved in 35% ACN in the presence of sodium acetate as a cationization agent was used as an optimized matrix. The nitrocellulose also suppressed matrix-derived ions and allowed those low molecular mass components to be observed which would otherwise be obscured, such as low-carbon-number TAGs. In addition, better shot-to-shot and sample-to-sample reproducibility was achieved.

MALDI-TOF MS of 14 different edible oils was combined with linear discriminant analysis (LDA) to show the applicability of this approach for authentication [51]. The DHB matrix in acetone containing 0.25% trifluoroacetic acid (TFA) was spotted onto the target for crystallization before adding the oil sample diluted in chloroform. Mass spectra characterized by the presence of sodiated ions were acquired in the reflector positive ion mode with a relatively good shot-to-shot reproducibility. Relative TAG contents (peak intensities) were then used for LDA. The rate of the correct classification was over 93%. Similarly, a DHB matrix in 90% methanol was utilized for the differentiation and classification of vegetable oils and animal fats (butter, lard) [59]. The mass and abundance values of the detected sodiated TAG ions (main isotopic peaks) were used for a cluster analysis discriminating the analyzed oils, namely, the animal fats because of their higher content of C16 fatty acids. The relative intensities of the ions provided predictions of the fatty acid composition, which appeared in a good agreement with literature data from different analytical methods. Edible oils are used in human society on a daily basis for cooking and food preparation. Different oils are characterized by different properties, nutrition values, and prices. As a consequence, adulterations have frequently been reported [13]. The problem of EVOO adulteration with hazelnut oil, which shows a high degree of similarity as regards to the profile of TAGs, sterols, and fatty acids, has been addressed by MALDI-TOF MS of phospholipids [13]. Their low content in EVOO precluded a direct analysis, and thus a pre-concentration treatment was necessary. This involved selective extraction with ionic liquid composed of tributylamine and CHCA, which was also applied as a matrix for subsequent MALDI measurements. Phospholipid signals were observed in the *m/z* region of 730–850. A phospholipid peak at *m/z* 786.5, attributed to dioleoylphosphatidylcholine, was a typical marker of hazelnut oil and could be detected in EVOO down to 1% *v/v*. Coupling this ionic liquid matrix-based approach with statistical data processing methods such as unsupervised hierarchical clustering, PCA and Pearson’s correlation analysis allowed adulterations of EVOO with corn oil down to 0.5% *v/v* to be recognized [60]. Simulated adulterations of EVOO, either by sunflower oil or refined olive pomace oil, were studied by MALDI-TOF MS of TAGs using a DHB matrix dissolved in THF in the presence of sodium trifluoracetate [11]. In parallel, the EVOO samples were also characterized by spectrophotometric measurements at 232 and 270 nm to detect the presence of hydroperoxides/conjugated dienes and carbonyls/conjugated trienes, respectively. A combination of MALDI-TOF MS profiling and PCA plots detected adulteration down to 1%, regardless of the geographical origin of EVOO. MALDI itself was successful at a rate of 10%, whereas the spectrophotometry could not recognize adulteration rates lower than 20%.

**Figure 2:**
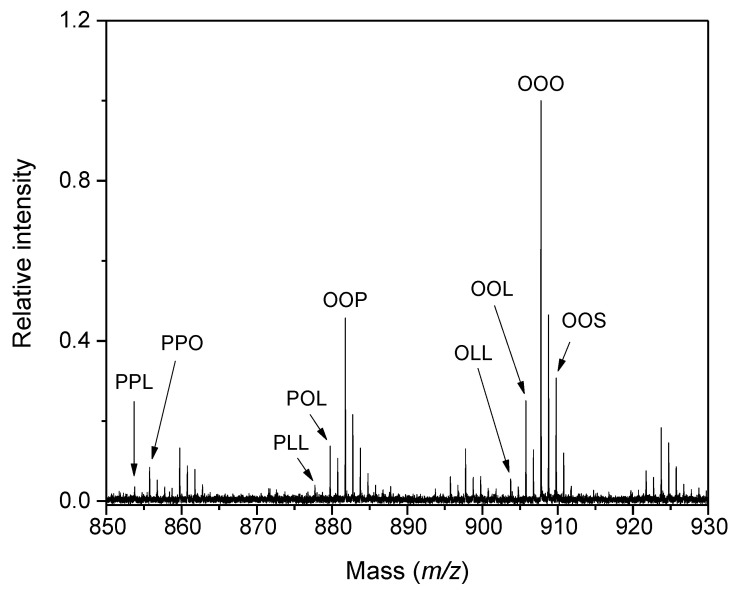
MALDI-TOF MS of TAGs in olive oil. This commercial virgin olive oil sample was measured using an ultrafleXtreme instrument (Bruker Daltonics). The depicted spectrum was acquired in the reflector positive ion mode with a stainless-steel target and DHB as a matrix according to De Marchi et al. [64]. Common TAGs were detected between *m/z* 850 and 930, mostly as sodium (but also potassium) adduct peaks. The labeling of selected sodiated ion peaks was done according to Calvano et al. [53]: L stands for linoleic acid, O for oleic acid, P for palmitic acid, and S for stearic acid. The content is author´s own work.

Another fraud problem residing in the illegal practice of selling refined waste oils (gutter oil) as a standard oil for alimentary purposes has appeared, particularly in Asian countries. A rapid and sensitive approach for the authentication of edible oils has been established with the direct application of oil samples on the target plate pre-spotted with DHB matrix for automated spectra acquisitions [61]. A reliable sample identification was achieved by comparing its profile spectrum (individual peak intensities were normalized to the total intensity) with a database of reference spectra using statistical analyses (PCA, hierarchical clustering). The procedure was successfully applied to pure vegetable oils, animal fats, mixed oils, as well as gutter oils. An olive oil adulteration by canola oil could be detected down to 2% [61]. Similarly, coupling MALDI-TOF MS with a multivariate statistical analysis of the spectral data allowed TAGs to be quantified and to determine blending ratios in binary, ternary, or even quaternary oil mixtures [62]. PLS regression extracts the most important information from complex data to build up reduced-dimension models. Oil blends were prepared by mixing pure vegetable oils (olive, perilla, rice bran, and sunflower oils) in different ratios for training and test sets up to 286 samples for the ternary mixtures. The limit of detection of olive oil in sunflower oil was 0.9% by calculation. The most abundant TAG signals for olive oils (Figure 2) were provided by triolein (OOO), *m/z* 907.8, and dioleoylpalmitin (OOP), *m/z* 881.8, whereas those at *m/z* 901.7 (trilinolein, LLL) and 903.7 (dilinoleoylolein, OLL) were most pronounced in sunflower oil spectra. The relative errors after training-set optimizations were around 20% for the low-abundance compositions (~5%) of ternary mixtures. In a comparison with GC, the MALDI-based approach resulted in comparable quantitative results, but more rapidly, and without the need for derivatization and column separation [62]. A different approach, which combined MALDI-TOF MS (with DHB as a matrix dissolved in acetone) and cosine similarity analysis of TAG spectral profiles, provided rapid and quantitative comparison and differentiation of 15 edible oils, lard, and adulterated samples as solutions made in chloroform. The cosine analysis resided in calculated score values from pairwise comparisons of reference and target spectra. In addition, the authors introduced a network visualization of TAG similarity correlations for a systematic classification of oil samples [63].

Pomegranate (*Punica granatum*) oil is rather rare but a health-beneficial product. GC–MS and MALDI-TOF MS were employed to characterize comprehensively its TAG profile and fatty acids [65]. Linolenic acid was found to be predominant (64–83%), and the major isomer of conjugated linolenic acids was identified as punicic acid by GC–MS. DHB in 90% methanol served as a MALDI matrix, and oil samples were dissolved in hexane. Positive ion MALDI mass spectra acquired in the reflector mode and *m/z* range of 450–2400 indicated the characteristic presence of TAGs containing linolenic acid [65]. The same laboratory next applied the strategy of MALDI-TOF MS profiling with the DHB matrix for studying olive oils from different olive cultivars: Middle Eastern, Greek, Italian, and Spanish [66]. The most represented TAG was triolein followed by 1,3-dioleoyl-2-palmitoylglycerol. The relative content of fatty acids calculated from the abundances of individual TAGs as sodiated ions in the fingerprints was comparable with parallel GC–MS data. Significant variations were found among the analyzed cultivars [66]. In another study, MALDI-TOF MS data obtained with oil samples from two olive varieties grown in the same geographic area at different maturity stages revealed TAG profile changes [67]. The differences were statistically significant, as the ripening led to a decrease in palmitic acid and an increase in linoleic acid in the detected TAGs. PCA plots then clearly distinguished the two olive varieties as well as the maturity [67]. Parallel analyses of oil samples extracted from seeds of hybrid grape varieties involved ESI–MS and MALDI-TOF MS [64]. Sodium adduct peaks were observed and assigned to the respective TAG structures by the fragmentation patterns in ESI–MS/MS spectra. Relative peak intensities in the MALDI spectra (after a correction for the isotopic patterns) allowed for evaluations of the relative content of each detected TAG. Unsaturated TAGs represented 70–80% of the total amount, and no significant difference between red and white varieties was found.

MALDI-TOF MS was used to acquire TAG profiles in a study that addressed the influence of different TAG compositions on the formation of structural crystal polymorphs of milk fat during isothermal crystallization at 20 °C [68]. DHB was used as a matrix and NaCl as a cationization agent. The analyzed milk fat samples differed in their 34–38/52–54 TAG ratios (the numbers represent the carbon atoms counts), as initially determined by GC. The contents of the relevant 34–38 and 52–54 TAGs were calculated from the relative ion abundances in the mass spectra. In parallel, milk fat crystals were subjected to X-ray diffraction for monitoring the crystal polymorphs formed. The formation of α polymorphs was promoted by saturated TAGs 34−38, whereas unsaturated TAGs 52−54 promoted the formation of β polymorphs [68]. Similarly, TAGs were profiled to evaluate differences between summer and winter cow’s milk, but in this case with THAP as a matrix. Interestingly, the abundance of polyunsaturated TAGs (>50 carbons) was higher in summer milk fat. This is in accordance with the known fact that cow’s feed (e.g., fresh grass and pasture in summer and silage in winter) largely accounts for differences in the composition of fatty acids in milk fat [69]. The above two studies utilized Pearson’s correlation-based hierarchical clustering analysis to process the determined TAG relative intensity data for the analyzed samples [68,69].

As indicated by the provided literature survey, MALDI-TOF MS profiling of TAGs or phospholipids is performed particularly to determine the components of oil/fat samples, which is very helpful to detect possible adulterations, e.g., when EVOO is mixed with common and cheap vegetable oils, or to obtain clues to solve biological and technological problems. The measurements are run in the reflector mode corresponding to the low molecular weight of the lipid compounds. TAGs are measured directly (e.g., diluted in hexane or chloroform), whereas phospholipids require a pre-concentration treatment for samples with their low content. Quantitative information is obtained from the relative ion intensities. Combining MALDI-based data with multivariate statistical analyses allows one to quantify TAGs in complex mixtures such as ternary or quaternary blends of oils.

## 4. Wine

**Figure 3:**
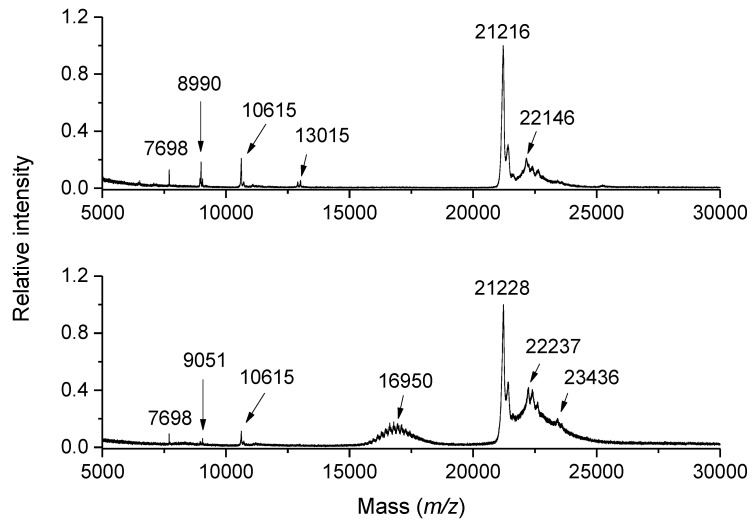
MALDI-TOF MS of white wine. All measurements were conducted using a Microflex LRF20 instrument (Bruker Daltonics), and the mass spectra were acquired in the reflector positive ion mode with an MSP BigAnchor 96 BC target and SA as a matrix (10 mg/mL in ACN:0.1% TFA, 1:1, *v/v*). The top panel refers to a partially fermented grape juice (Federweisser) after dialysis against water, while the bottom panel spectrum comes from a dialyzed and ultrafiltered Sauvignon blanc wine. Each sample (1 µL) was mixed with 1 µL of the matrix solution on the target and left to dry and crystallize prior to the measurements. The content is author´s own work.

The chemical components of wine range from simple organic molecules such as alcohols, monosaccharides, organic acids, esters, and flavonols to polymeric condensed tannins, polysaccharides, and proteins. Red wines are characterized by the presence of colored anthocyanins [70]. Each wine has a unique pattern of organic compounds. The most common analytical approach to resolve and detect these compounds is liquid chromatography coupled to mass spectrometry, i.e., LC–MS. Due to its simplicity and sensitivity, MALDI-TOF MS has been shown to be very applicable for the rapid analysis of proteins in white wine [71]. The analyzed wine samples were mixed with SA matrix without any pre-treatment or after a series of precipitation steps to purify proteins and increase their concentration. Protein peaks recorded between *m/z* 5000 and 30,000 indicated, among others, the presence of glycoproteins and displayed differences resulting from the use of different grape varieties. Figure 3 shows MALDI-TOF spectral profiles of different white wines. The most striking is the presence of thaumatin-like proteins (*m/z* 20,000–24,000) [72] and the *O*-glycosylated protein seripauperin 5 (Pau5; the peak cluster centered at *m/z* 17,000) [73]. Based on the protein content, such profiles can be measured directly or after a concentrating pre-treatment, e.g., using an ultrafiltration (Šebela, M., unpublished results).

A fast fingerprinting method utilizing DHB as a MALDI matrix allowed the chemical composition of 20 different Italian red wines to be compared [74]. Various chemical compounds (*m/z* range 73–1760), including red wine pigments, were simultaneously registered in the acquired mass spectra, showing clear qualitative and quantitative differences among the studied wines. The measurements were performed directly without any sample pre-treatment or enrichment procedures. Similarly, phenolic acids and resveratrol in red wines were profiled by LDI-TOF MS [75]. Their chemical structure, which is similar to the commonly used MALDI matrices such as CHCA, DHB, and ferulic acid (FerA), the latter being among the detected natural wine components, allowed for efficient ionization while avoiding matrix-related interfering signals. Nevertheless, first the wine samples had to be processed by a solid phase extraction. Differences in the content of these compounds in Cabernet Sauvignon and Merlot wines originating from Chile and Australia were quantified from the signal-to-noise ratios of the corresponding spectral ions.

Reproducible MALDI-TOF fingerprint spectra of grape seed proteins were obtained after powdering the material in liquid nitrogen, defatting by hexane, and dialysis, followed by an optimized protein extraction in 0.1% TFA [76]. The spectra were acquired in the linear positive ion mode with DHB as a matrix. This approach was applied to evaluate the potential of the method for the rapid differentiation of grapevine varieties. For example, a peak with *m/z* 6110 was found to be a marker of the Raboso Piave variety, whereas that with *m/z* 4306 indicated the Malvasia Nera variety. The latter variety additionally showed specific peaks around *m/z* 14,000. Important parameters from the point of view of analytical chemistry for the accurate MALDI-based fingerprinting of wine include the selection of the matrix, the spectrum processing procedure (peak intensity normalization), and the number of individual and technical replicates. This was addressed in a study with Spanish wines measured using a CHCA matrix [77]. The obtained spectral data (*m/z* 40–1500) were analyzed by different artificial intelligence algorithms, and accuracy values appeared in the range of 88–95%. A minimum of five bottles of each wine and three spot replicates per bottle were recommended for a correct classification.

Direct measurements with wine samples proved clearly to be useful for the molecular profiling of proteins as well as low molecular weight compounds (e.g., pigments in red wine and organic acids). The obtained profiles can be applied for the rapid differentiation and classification of wines. In addition, marker proteins may have interesting biological and technological significance. The absence of Pau5, for example, was correlated with the gushing phenomenon of sparkling wines. As a consequence, foam stabilizing properties of the protein have been assumed [73]. The measurements with proteins may require a volume reduction and concentration of the sample, depending on their varying content.

## 5. Beer

Chemical analyses of beer have recently been thoroughly reviewed as regards to the initial ingredients for brewing and the final product as well. Beer contains more than 3000 different compounds in solution, including sugars, proteins, ions, organic acids, and polyphenols. Amino acids, peptides, and proteins are typically analyzed by LC, but capillary electrophoresis is also applicable [78]. MALDI-TOF MS is a good choice for monitoring relevant microorganisms such as brewing yeasts. A spectral database of 52 *Saccharomyces* strains covering 32 top-fermenting strains (*S. cerevisiae*), 13 bottom-fermenting strains (*S. pastorianus*), and 7 *S. cerevisiae* var. *diastaticus* strains (a beer spoilage variety) was prepared following a protocol based on the extraction of proteins from cultivated cells using 70% formic acid and ACN prior to MALDI-TOF MS with a CHCA matrix. The database was successfully validated with test strains and allowed for a highly accurate assignment, not only as regards to the species but also to a specific beer type of preferential use [79]. In spite of the antimicrobial properties of beer, spoilage microbes may persist after the brewing process and deteriorate the product quality or release toxic metabolites such as *Fusarium* mycotoxins [78]. A MALDI biotyping strategy based on measuring protein profiles with extracts of intact cells grown on agar plates or in a liquid medium and searches across a database of reference spectra has been established for the simple, fast, and reliable identification of beer spoilage microbes [80]. The authors used a model system consisting of brewing yeasts, a wild yeast, and several spoilage bacteria to extend their commercial library of reference spectra of microorganisms. The applicability of the procedure was verified by identifications from spiked beer samples and then tested with real brewery samples in the form of spread agar plates (this was a kind of enrichment necessary to achieve a reasonable sensitivity). The obtained identification scores allowed for the reliable confirmation at the species or at least genus level (e.g., *Enterobacter*; lactic acid fermentation bacteria; and *Candida*, *Pichia*, *Dekkera*, and *Rhodotorula* yeasts).

**Figure 4:**
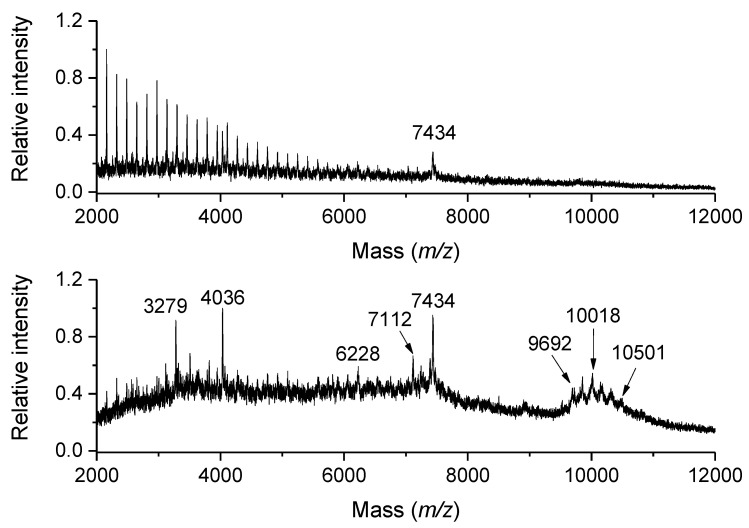
MALDI-TOF MS of beer. This sample of a non-pasteurized Czech lager beer was measured using an ultrafleXtreme instrument (Bruker Daltonics). The depicted mass spectra were acquired in the linear positive ion mode with a stainless-steel target and DHB as a matrix according to Šedo et al. [82]. The top panel shows a series of maltooligosacharides (*m/z* 2160–7365, sodium adduct peaks, with a regular mass difference of 162 units) and was acquired from the central part of the sample spot. The bottom spectrum shows signals of beer proteins. It was acquired from the rim of the sample spot. Interestingly, the signal cluster between *m/z* 9692 and 10,501 refers to the non-specific lipid transfer protein 1 (LTP1) from barley and its glycated forms [81]. The content is author´s own work.

Water-soluble proteins extracted from grains and malt were compared between two barley varieties using sodium dodecylsulfate polyacrylamide gel electrophoresis and MALDI-TOF MS protein profiling [81]. Proteins in beer and beer foam were also analyzed in this way. 2,6-Dihydroxyacetophenone (DHAP) performed best when comparing three different matrices (the others were SA and THAP) to achieve protein peaks of the highest intensity. The use of DHB as a matrix also proved efficient for a direct beer analysis by MALDI-TOF MS [82]. Small aliquots of beer were premixed with the matrix prior to on-target co-crystallization. The resulting crystal pattern consisted of a central area with small crystals surrounded by large needle-shaped crystals on the rim of the sample spot. Interestingly, maltooligosaccharides with molecular masses up to 5 kDa were separately ionized from the central part, whereas the border crystals allowed peptide and protein spectra to be acquired (Figure 4). Such reproducible protein profiles were found to be applicable for a hierarchical tree clustering of the analyzed beer samples, which distinguished beer of the same brand from different breweries. The same group performed a related study on malting barley varieties and their software discrimination based on protein profile spectra [83]. They analyzed powdered barley grains directly with FerA as a matrix optimized in a working solution containing 17% formic acid. This arrangement and measurements in the linear positive ion mode led to the successful recording of discriminating protein signals at around *m/z* 45,000.

The 2,6-positional isomer of dihydroxybenzoic acid was found to be superior to the commonly used 2,5-isomer (i.e., DHB) for MALDI-TOF MS analysis of beer oligosaccharides detected as potassium or sodium adducts [84]. Another isomer, 2,4-dihydroxybenzoic acid, was employed successfully for the profiling of maltooligosaccharides in beer and wort as well as in a study of the branching pattern of beer isomaltooligosacharides isolated by HPLC [85]. Highly reproducible maltooligosaccharide profiles of beer (upon a 100-fold dilution) were recently acquired using a reactive matrix compound, 2-phenyl-3-(*p*-aminophenyl)acrylonitrile (PAPAN), a derivative of α-cyanocinnamic acid reacting at the reducing end of oligosaccharides, in the presence of sodium ions [86]. The results were evaluated by constructing a dendrogram for different beer brands, indicating the large discrimination power of the method. Furthermore, because of its sensitivity at the level of femtomoles, the method offers applicability in high-throughput screening studies. Both maltooligosaccharide and protein profiles measured with beer samples using MALDI-TOF MS are applicable to distinguish different beer brands or, potentially, the same brand produced by different breweries. A clear advantage of this approach resides in the possibility of direct spectra acquisitions without any pre-treatment.

## 6. Other Food Products

Gliadins are a complex group of proteins containing immunogenic epitopes, which may trigger human diseases such as celiac disease and food allergy. Common assays rely on the use of antibodies [87]. A procedure for the classification of wheat varieties by MALDI-TOF MS profiling of gliadins utilized spectral peaks in the *m/z* range of 14,000–45,000 [88]. The proteins were extracted from the flour by 70% ethanol, and the extracts were mixed with an excess of the SA matrix solution prior to deposition onto the target plate. The acquired data (linear positive mode) were processed with an artificial neural network. At least 20 spectra for each variety represented a training set, and another five were used for testing. The procedure was found to be reliable, fast and robust; the accuracy of classification was 97%, and the results were independent of the storage of flour and extracts as well as a possible exchange of instrument operators [88]. Recently, MALDI-TOF MS was compared with reversed-phase high performance liquid chromatography (RP-HPLC) for analysis of gliadins from Chinese Spring (CS) wheat, which indicated advantages and limits of the methods [89]. These proteins were isolated from a standard CS wheat and its aneuploid lines with altered gliadin composition and measured with SA as a matrix using the double layer deposition technique. The optimized sample preparation protocol led to 13 gliadin peaks observed in the mass spectra acquired in the linear positive mode (9 peaks at 29–40 kDa, 2 peaks at 40–44 kDa, and 2 peaks at 50–57 kDa). They were assigned to α/γ-, ω-1, 2-, and ω-5 gliadins, respectively. Because of the resolution and low mass differences, some peaks referred to more coding genes at different chromosomes. MALDI-TOF MS appeared to be suitable for the rapid screening of specific gliadins (e.g., ω-1, 2-, and ω-5). A similarity in molecular masses of α- and γ-gliadin limits the applicability of MALDI, whereas HPLC has a broad scope for checking overall changes.

MALDI-TOF mass spectra acquired in linear positive ion mode were used to evaluate extracts of industrial soy protein isolates, which differ in their composition and degree of hydrolysis [90]. In the food industry, different isolates yield food products with different properties, such as taste and consistency, which justify the need for a rapid analysis. Two major storage proteins are present in soybeans: glycinin and β-conglycinin. The measured samples were treated by reducing conditions during extraction to detect subunits of the complex proteins, which disappear upon hydrolysis, which influences both the performance and functionality of the isolate. The matrix used was a mixture of DHB and 2-hydroxy-5-methoxybenzoic acid, namely, super DHB [90]. In another study, soy flour from dried and germinated beans was analyzed by MALDI-TOF MS for the presence of bioactive peptides with nutraceutical functions [91]. The analysis included flour extracts and their hydrolysates prepared with an alcalase protease from *Bacillus licheniformis*. The treatment resulted in differences in the spectrum of peptides originating particularly from β-conglycinin and glycinin. Other bioanalytical methods demonstrated an increased antioxidant capacity of the flour upon germination and potential anti-inflammatory effects. A MALDI-based MS profiling of small proteins was performed to distinguish transformed and untransformed potato tubers [92]. The genetically modified potato was a result of antisense technology for inhibiting the expression of a gene, which regulates the transition from dormancy to sprouting. Several signals detected using SA as a matrix were found to be characteristic of the transformed potato (e.g., *m/z* 4373 and 4932). The potato extracts were prepared by processing apical eyes, and the whole tubers were found to be unsuitable to recognize differences. Two peaks (*m/z* 4292 and 4317), which disappeared in comparison with the control line upon transformation, were attributable by mass to possible protein products of G1-1 gene expression. The acquired MALDI-TOF MS data were consistent with reverse-transcriptase polymerase chain reaction analyses to determine the decreased transcript level of the studied gene [92].

MALDI-TOF MS has been included among other modern methods (immunoassays, DNA-based methods, etc.) for meat authentication assessments [15]. Proteomic approaches are applied in studying differences in peptides and proteins related to the proteolytic changes, which occur during the processing and aging of meat. Exogenous proteins that can potentially be added to meat such as soybean proteins or muscle tissue proteins of another species (e.g., chicken versus turkey) are detectable via specific tryptic peptides [15]. Fish represents an important food in the diet of humans. Protein extracts from the muscle tissue were subjected to molecular profiling by MALDI-TOF MS to discriminate unambiguously 25 different fish species, including, for example, seabass, seabream, hake, halibut, sole, and Atlantic cod [93]. Signals of marker proteins were found at *m/z* 11,200–12,100 using CHCA as a matrix when measuring in the linear positive ion mode. The marker proteins were identified as fish prolamins after LC separation of the extracts followed by MALDI peptide mass fingerprinting and/or ESI–MS/MS of the corresponding fractions. Interestingly, the proteins were stable enough to be useful as marker molecules for heat-treated samples, as well as indicating their possible applications for the authentication of cooked products [93]. Similarly, species-specific protein patterns of common food fish such as the Atlantic or Pacific cod, trout, and catfish were acquired by MALDI-TOF MS and used to build a reference library (54 species of 24 families). The aim was to develop a tool applicable to detect mislabeling and fraud with respect to fish meat products as a rapid alternative to molecular DNA methods [94]. Fillet meat samples were treated for protein extraction, and the extracts were measured using a CHCA matrix. Validations were done by PCR and DNA sequencing. The database was tested with 118 samples providing a 96% rate of positive identifications at the species level. In addition, possible fish contamination by microbes was simulated by adding *Escherichia coli* biomass into the extracts. The addition of 1% by weight still allowed for the correct identification [94]. Another rapid screening method based on MALDI-TOF MS was developed for the evaluation of trout freshness by determining the exact storage day after fishing [95]. The measured biological material was sampled from the vitreous fluid of fish eyes on different days of storage after fishing. Mass spectra were acquired with CHCA as a matrix in the linear positive mode (*m/z* 2000–20,000). There were four marker peaks, and the resulting recognition capability for models comparing day 0 to the others (3–11) ranged from 96.2% to 98.8%. The overall external validation with an independent sample group provided correct identification rates of around 90%. However, distinguishing among days at the end of the storage period was a serious limitation. However, this approach could represent a good alternative to the established sensory and spectroscopic methods, which are negatively influenced by the natural variability in color of fish tissues.

MALDI MS imaging of dry-cured Istrian ham was shown to be suitable for batch quality control of this meat product [96]. The most critical optimizations in the development of the experimental protocol were those of sample embedding prior to making tissues slices and their washings to reduce the content of salts and lipids. Mass spectra were acquired in the linear positive ion mode with CHCA as a matrix. Peptides (originating particularly from the troponin C muscle protein) were registered in the *m/z* range of 1200–2400, and differences were found in their spatial and intensity distributions among four ham samples from the same production batch. This indicated a different level of proteolysis that had occurred during the maturation of the product. The cleavage sites mapping resulted in the finding that serine endopeptidases are largely involved in the proteolysis process [96]. The applicability of MALDI-TOF MS combined with chemometrics for the successful differentiation of frozen–thawed duck fatty livers (i.e., “foie gras” in French) from fresh ones was demonstrated recently [97]. The liver samples were obtained from more places of origin, with a different weight range, fresh and stored in a fridge up to 14 days, or frozen–thawed and then stored at 4 °C up to 14 days. Protein profiles of different liver extracts (*m/z* 1000–20,000) were acquired in the linear positive ion mode using CHCA as a matrix. The data were first processed by PCA and then by a supervised classification model (using three different algorithms) with a recognition capability of around 90%. Similarly, the same laboratory analyzed partially purified blood plasma proteins from pigs slaughtered to produce a French cooked ham to find predictive clues for distinguishing a standard ham product from that with a defect manifested by holes in the muscle structure. The obtained results were comparable as regards to the classification accuracy (85%) and other reliability parameters to those obtained by infrared spectroscopy [98]. When the data were combined and analyzed together by a neural network, the classification accuracy reached 100%. A simple extraction, which is based on the homogenization of muscle tissue in the presence of silica beads in acidified 50% ACN (as developed previously for the classification and authentication of mozzarella and feta cheeses [99]), was applied to build a database of reference “meat” protein spectra for 265 species (mammals, birds, and reptiles). The database was then successfully validated with numerous meat samples, and the identification rates were typically around 85–95% [100].

The MALDI-TOF MS profile spectra of peptides and proteins extracted from powdered green coffee originating from different plantations worldwide were measured with a saturated SA matrix [101]. The data showed peak representation differences between *Coffea arabica* and *C. robusta*. A clear variability in the relative abundances of the detected peaks (*m/z* 2000–14,000) was observed for multiple samples of the same type. This was utilized in hierarchical clustering analyses [101]. MALDI-TOF MS of Hawaiian honey from different production sites was performed in linear positive mode to construct a database of protein profiles transformed into barcodes [102]. Common signals appeared typically between *m/z* 4500 and 6500, whereas the region of *m/z* 8500–10,000 provided some differential peaks. The database was successfully used to assess the geographical origin and authenticity of 38 commercial honey samples. Low correlation coefficients were found by comparing the peak lists from the Hawaiian honey database with those obtained for distant geographic regions of honey origin (e.g., North America and Asia), which was obvious also from data visualization by PCA.

MALDI-TOF MS in a low-molecular-weight window of *m/z* 100–1000 was proven to be useful to classify chocolate samples according to their cocoa content [103]. Chocolate extracts prepared by fractionating extraction finished with 70% formic acid, and ACN was measured in the reflector positive mode with a saturated CHCA matrix. The profile spectra for each chocolate with a known cocoa content (despite the presence of numerous matrix-related signals), acquired in replicates, were processed into a standardized reference spectrum to build a library. The reference spectra were clearly clustered according to the corresponding cocoa content. The library was then used with different chocolate extracts as test samples with accuracy values up to 78%, showing a higher reliability for samples with a higher cocoa content. In another study, the cocoa content was analyzed by MALDI mass spectrometric imaging of catechin/epicatechin (*m/z* 291) as a marker in chocolate imprints [104]. It has been shown by relative quantification based on pixel intensity that the marker compound is directly proportional to the declared cocoa content value.

The application of MALDI-TOF MS-based profiling has been shown to be universal for many kinds of food products. According to need, proteins and peptides or low-molecular-weight metabolites are analyzed as target compounds. The most common matrices for such experiments are SA and CHCA, respectively. The presence of marker peaks combined with quantification, PCA, or supervised statistical analyses is very applicable for authentication, quality control, as well as differentiation and classification of samples.

## 7. Summarizing Tables

The reported combinations of MALDI-TOF MS profiling with a peak intensity-based quantification of marker compounds or statistical data evaluation for sample differentiation and classification have proven efficient, reliable, and very promising for routine analyses of food and beverages. The involvement of this strategy utilized many advantages of biomolecular MALDI-TOF MS, including its speed, simplicity, versatility, minimum sample preparation needs, and relatively high tolerance to salts. The studied food or beverage samples were measured directly or after a short and undemanding pre-treatment, for example, when phospholipids and phosphopeptides were enriched and then employed as markers to characterize olive oil [13] and milk [37], respectively. The preparation of sample spots on the target plate requires optimization by selecting the most suitable matrix compound and sample deposition technique. Table 1 shows an overview of the matrices that have been used in relevant applications. In addition to ensuring efficient ionization yielding the highest peak intensities (related to the sensitivity of the analysis), it is important to allow the sample-matrix co-crystals to grow into a homogenous layer to achieve the best possible shot-to-shot and sample-to-sample reproducibility during spectra acquisition. This is appreciated especially in quantitative studies. DHB is a clear example. When dissolved in a volatile organic solvent (e.g., hexane, chloroform, acetone, or THF) it yields a layer of small and homogenous crystals upon evaporation (compared to the “needle-shaped” crystals from solutions in water). When using DHB, edible oil samples are deposited onto the matrix crystal layer [51], premixed with the matrix prior to deposition [64], or applied onto matrix crystals covering a thin layer of nitrocellulose [58]. Alternatively, oils can be deposited directly by a pipette tip onto the top of a matrix layer at the target plate to make a lipidic thin layer [62]. The DHB matrix is optionally doped with sodium acetate/trifluorocetate salts for a better analyte ionization [11,58]. The homogenous layer of sample-matrix co-crystals is always a good premise for automated and high throughput measurements.

The biggest potential of MALDI-based food resides in their availability for immediate and rapid use, which is helpful for detecting fraud and adulterations. Another motivation for these experiments resides, for example, in looking for specific molecules, evaluation of sample components, freshness assessment, quality control in technological processes, and authentication in the sense of geographical origin. A general survey of the use of MALDI-TOF MS is provided in Table 2. It is an excerpt from the published reports covered in this review and summarizes targeted molecules, experimental conditions, data processing strategies, and application purposes. Protein and peptide markers have been studied for milk and milk products. Caseins and whey proteins can be identified by their specific molecular mass, and thus the recognized mass difference indicates the presence of milk originating from a different animal source [30]. The presence of other peptides than expected is indicative of an adulteration [36]. Furthermore, differences in the level of peptide markers obtained by quantitative experiments suggest a treatment resulting in increased proteolysis, which is relevant for both the milk storage period [43] and cheese ripening [41]. Similarly, the presence of specific TAGs or their different relative ratios allows for the identification of various edible oils and can detect their adulteration, which is a common problem, especially in the case of EVOO [11,13]. Maltooligosaccharides in beer show different profiles related to the alcohol content and reflecting the activity of fermenting enzymes [85]. The acquisition of MALDI-TOF MS profile spectra does not need to be accompanied by the identification or quantification of specific marker biomolecules. Comparing the peak position and intensity patterns is the basis for their differentiation, which is applied for classification/regression purposes or to detect fraud and adulterations. Achieving such goals requires the use of software tools for advanced data analysis. The most common is PCA, but supervised statistical methods with training and testing datasets are recommended for large numbers of samples. Proteins and/or peptides have been applied to discriminate milk, flour, meat, beer, honey, etc., whereas TAGs and phospholipids have been used for vegetable oils (Table 2 and references therein). Finally, MALDI-TOF MS offers researchers and instrument operators multistep optimizations (from sample preparation and deposition on the target plate to the spectra acquisition and data analysis procedures) to develop a protocol tailored according to their needs in order to afford the best performance on the way to achieving their analytical goals.

## 8. Conclusions

This review article shows the extent of using MALDI-TOF MS profiling of biological molecules in food and beverage samples. The studied material spans from cheese and meat to honey and chocolate or from milk to wine. Sample preparation seems to be a crucial point for successful measurements and the obtaining of relevant data. The easiest way is a direct measurement or including just a dilution or simple extraction (e.g., in the case of milk, vegetable oils, wine, or beer). It is highly desirable to perform only a single-step extraction, which has been reported, for example, for cheese, flour, meat, and honey. According to my opinion, especially those methods that do not require multistep and time-consuming sample preparation procedures have become very promising and could easily be transferred into practical applications. Enrichment strategies for specific molecules such as peptides/phosphopeptides are acceptable as long as they do not lengthen the overall protocol too much. This is the case for efficient affinity purification steps, which have been reported so far and can be further elaborated or developed by an analogy for different sample types. Another important aspect, which needs to be considered for routine MALDI-based analyses in industrial or state authority quality control and assurance laboratories, is user comfort for operators recruited from the staff of technicians. Importantly, most experimental demands in molecular profiling of food and beverages can be addressed on benchtop linear MALDI-TOF mass spectrometers. The use of instruments equipped with the reflector mode is definitely advantageous for measuring low molecular weight compounds such as peptides, TAGs, and phospholipids because of the higher resolution available, which is helpful when quantitative data are necessary for a particular marker ion species. On the other hand, it is not necessarily needed for spectral barcoding or other data processing schemes (bioinformatics) utilizing the whole spectrum as a sample fingerprint.

Great potential can be seen in the near future for the possibility of tracking more types of compounds in a single sample. For example, proteins, peptides, and TAGs were shown to be all measurable in diluted milk using different matrices in optimized protocols. Moreover, sample fractionations based on ultrafiltration and sequential extractions are at hand. The question of which data analysis approach should be chosen and which statistical procedure followed is very important and depends on the aims. Large data sets, in my opinion when more than a hundred samples are processed, require supervised multivariate statistical analyses and building more models to be evaluated and validated prior to introducing a final standardized protocol. The availability in the market for instrumentation for MALDI-TOF MS profiling with the aim to solve food composition, safety, and authenticity issues is announced by the producers. MALDI-TOF mass spectrometers represent analytical tools that are advantageous over conventional techniques (e.g., liquid chromatography) because of their high throughput and speed. They also offer much higher accuracy and specificity. Molecular profiling strategies in connection with intact cell MALDI-TOF MS of microorganisms have found their widespread practical application in clinical, veterinary, and hygiene laboratories. Hopefully, their use for food and beverage inspection will also expand. It is clear that automated measurements are needed for reaching high throughput profiling. They are certainly feasible on current instruments.

## Figures and Tables

**Table 1:** An overview of the MALDI matrices that have been used for food and beverage analyses.

Matrix Compound	Application Examples and Solvents Utilized for Dissolution (in Parentheses)	References
CHCA	Milk, peptides (30% ACN + 0.1% TFA in water); oils, TAGs (ACN:THF, 7:1, *v/v*); meat, proteins (2.5% TFA in 50% ACN); honey, peptides (30% ACN + 0.1% TFA in water)	[38,56,95,102]
CHCA + tributylamine	Oils, phospholipids (equimolar CHCA and tributylamine are dissolved in methanol, which is then evaporated)	[13,60]
CClCA	Milk, peptides (ACN:water, 2:1, *v/v*, with 0.1% TFA)	[30]
DHAP	Beer, proteins (ACN:water, 1:1, *v/v*)	[81]
DHB	Oils, phospholipids (methanol:ACN, 1:1, *v/v*); kefir, phosphopeptides (ACN:0.1% TFA, 3:2, *v/v*); oils, TAGs (acetone with 0.25% TFA); beer, oligosaccharides, and proteins (ACN:water:TFA, 50:49:1, *v/v/v*)	[13,42,51,82]
2,6-DHB	Beer, oligosaccharides (50% ACN + 0.1% TFA in water)	[84]
F20TPP	Oils, fatty acids (chloroform, sodium acetate as a dopant)	[57]
FerA	Flour/powdered grains, proteins (ACN:water: formic acid, 50:33:17, *v/v/v*)	[83]
PAPAN	Beer, oligosaccharides (methanol containing 5% acetic acid)	[86]
SA	Milk, proteins (ACN:0.1% TFA, 40:60, *v/v*); flour, proteins (ACN:methanol:water, 60:36:8, *v/v/v*)	[20,88]
Super DHB	Milk, lipids (70% ethanol); soy, proteins (not specified, recommended by instrument vendor: 30% ACN + 0.1% TFA in water)	[47,90]
THAP	Oils, TAGs (methanol saturated with NaCl)	[9]

**Table 2:** The use of MALDI-TOF MS for molecular profiling in food analysis. This table provides condensed but concise information regarding the sample type, targeted molecules, experimental conditions and sample treatment, data processing strategies, and application purposes.

Food/Beverage Type	Targeted Molecules	Acquisition Mode ^a^	Mass Range (Da)	Matrix and Deposition Technique	Sample Preparation Steps	Data Analysis	Application	References
Milk	Peptides	RP	700–4000	CClCA; mixed volume	Electrophoresis, in-gel digestion	Marker peaks, quantification	Milk adulteration	[30]
Milk	Proteins	LP	10,000–30,000	SA; mixed volume	Whey protein fraction	Marker peaks, quantification	Milk adulteration	[35]
Milk	Peptides, proteins	RP (peptides), LP (proteins)	500–5000 (peptides), 5000–20,000 (proteins)	CHCA (peptides), SA (proteins); mixed volume	Centrifugation, dilution (proteins), ultrafiltration (peptides)	Marker peaks, PCA, PLS regression	Milk adulteration, thermal treatment detection	[38]
Milk	Phospholipids, TAGs	LP	400–1000	Super-DHB; dried droplet	Dilution	PCA, prediction model	Milk adulteration	[47]
Cheese (mozzarella, Pecorino)	Proteins	LP	2000–25,000	SA; mixed volume	Centrifugation, extraction	Marker peaks	Cheese adulteration	[34]
Cheese (camembert)	Peptides	RP	600–3500	CHCA; dried droplet	Extraction, fractionation	Marker peaks, ANOVA, PLS-DA	Presence of bioactive peptides	[39]
Cheese (feta)	Proteins	LP	3500–40,000	SA; double layer	Extraction	PCA, PLS	Cheese adulteration	[40]
Cheese (cheddar)	Peptides	RP	Not specified	CHCA; technique not specified	Extraction	Marker peptide, quantification	Evaluation of bitterness	[41]
Kefir	Phosphopeptides	RP	600–5000	DHB; mixed volume	Hydroxyapatite enrichment	Marker peaks	Detection of bioactive compounds	[42]
Vegetable oils	TAGs	RP	10–1300	DHB; thin layer	Dilution	PLS-DA	Authentication	[51]
Vegetable oils	Fatty acids	RP	210–350	F20TPP; mixed volume	Saponification, Na^+^ as a cationizer	Marker peaks	Determination of TAGs composition	[57]
Vegetable oils	TAGs	RP	500–2000	DHB; thin layer	Direct measurement	Quantification, PLS regression	Quantification of TAGs, oil blending ratio determination	[62]
EVOO	TAGs, phospholipids	RP	400–1100	CHCA-based ionic liquid	Extraction with ionic liquid matrix	Marker peaks, quantification	Detection of adulteration	[13]
EVOO	TAGs, phospholipids	LP	300–1300	CHCA-based ionic liquid	Extraction with ionic liquid matrix	PCA, hierarchical clustering	Detection of adulteration	[60]
Milk fat	TAGs	RP	400–1000	DHB; mixed volume	Chloroform solution, Na^+^ as a cationizer	Hierarchical clustering	Evaluation of fat crystallization	[68]
Milk fat	TAGs	RP	400–1000	THAP; mixed volume	Chloroform solution, Na^+^ as a cationizer	Hierarchical clustering	Comparison of seasonal bulk milks	[69]
White wine	Proteins	LP	5000–30,000	SA; mixed volume	Dissolved precipitate	Marker peaks	Protein content evaluation	[71]
Red wine	Metabolites, pigments	RP	200–700	DHB; dried droplet	Direct measurement	Marker peaks	Differences in representation of compounds	[74]
Red wine	Metabolites, pigments	RP	40–1500	CHCA; mixed volume	Filtration	Artificial intelligence algorithms	Classification of bottled wine	[77]
Beer	Proteins	LP	2000–12,000	DHB; mixed volume	Direct measurement	Hierarchical clustering	Classification of bottled beer	[82]
Beer	Maltooligosacharides	RP	1000–3000	PAPAN; double layer	Direct measurement, Na^+^ as a cationizer	Hierarchical clustering	Classification of bottled beer	[86]
Powdered barley	Proteins	LP	2000–50,000	FerA; extract in matrix solution	Powdering of grains, extraction	Hierarchical clustering	Classification of malting barley varieties	[83]
Wheat flour	Proteins	LP	14,000–45,000	SA; mixed volume	Ethanol extraction	Artificial neural network	Classification of wheat varieties	[88]
Soy protein isolates	Proteins	LP	5000–100,000	Super-DHB; dried droplet	Extraction and reduction	Marker peaks	Composition and degree of hydrolysis	[90]
Soy flour	Peptides	LP	1000–20,000	Not specified (CHCA?)	Extraction and proteolysis	Marker peaks, ANOVA	Presence of bioactive peptides	[91]
Potato	Peptides and proteins	LP	3000–10,000	CHCA; mixed volume	Tuber/apical eyes, extraction	Marker peaks	GMO detection	[92]
Fish	Peptides and proteins	LP	2000–15,000	CHCA; dried droplet	Muscle tissue extraction	Marker peaks	Fish differentiation and authentication	[93]
Fish	Proteins	LP	2000–20,000	CHCA; dried droplet	Muscle tissue extraction	Spectral library, hierarchical clustering	Fish differentiation and authentication	[94]
Fish	Peptides and proteins	LP	2000–20,000	CHCA; mixed volume	Eye liquid, ethanol	Spectral library, hierarchical clustering	Fish freshness assessment	[95]
Dry-cured ham	Peptides	LP, imaging	700–4000	CHCA; sublimation	Cryo-sectioning	Intensity images, PCA	Quality control	[96]
Cooked ham	Peptides and proteins	LP	1000–10,000	CHCA; dried droplet	Blood plasma proteins—purification	PCA, neural network	Quality control	[98]
Foie gras	Peptides and proteins	LP	1000–20,000	CHCA; dried droplet	Protein extraction and purification	PCA, neural network	Freshness assessment	[97]
Coffee	Proteins	LP	2000–20,000	CHCA; sample suspension in the matrix solution	Dialysis, grinding, extraction	Hierarchical clustering	Classification of coffee beans	[101]
Honey	Peptides and proteins	LP	1000–10,000	CHCA; mixed volume	Extraction	Spectral barcodes, PCA	Authentication, geographical origin	[102]
Chocolate	Catechin/ epicatechin	RP, imaging	100–500	CHCA; spray	Imprinting	Intensity images	Cocoa content (quality control)	[104]

^a^ The abbreviations LP and RP stand for linear positive ion mode and reflector positive ion mode, respectively.

## Data Availability

Not applicable.
